# The HCG11/QKI5 Axis Regulates Endothelial Angiogenic Adaptation During Chronic Cerebral Hypoperfusion

**DOI:** 10.1002/cns.71042

**Published:** 2026-07-22

**Authors:** Xueqiao Jiao, Rui Li, Lulan Li, Xiao Wu, Lu Wang, Fuxia Yang, Fangfang Zhang, Jia Liu, Xunming Ji, Xiuhai Guo

**Affiliations:** ^1^ Department of Neurology Xuanwu Hospital, Capital Medical University Beijing China; ^2^ Department of Geriatrics, Shanghai Tenth People's Hospital Tongji University School of Medicine Shanghai China; ^3^ Hypoxia Conditioning Translational Laboratory of Clinical Medicine Capital Medical University Beijing China

**Keywords:** chronic cerebral hypoperfusion, intracranial atherosclerotic stenosis, lncRNA HCG11, QKI5, vascular compensation

## Abstract

**Aims:**

Chronic cerebral hypoperfusion drives the progression of intracranial atherosclerotic stenosis (ICAS) and increases ischemic risk. Endothelial angiogenic adaptation initially preserves cerebral perfusion but deteriorates with disease progression. We investigated whether the HCG11/QKI5 axis represents a regulatory node with therapeutic potential in cerebral hypoperfusion.

**Methods:**

Circulating hypoxia‐ and angiogenesis‐related biomarkers were compared between asymptomatic and symptomatic ICAS patients to reflect different progression stages. Endothelial function was assessed in oxygen–glucose deprivation models following HCG11 overexpression or QKI5 knockdown. A rat model of chronic cerebral hypoperfusion was used to evaluate temporal changes in vivo.

**Results:**

Pro‐angiogenic factors were elevated in asymptomatic ICAS but reduced in symptomatic patients, indicating progressive impairment of vascular compensation. HCG11 overexpression enhanced endothelial proliferation and tube formation, whereas QKI5 silencing attenuated these responses. These effects were associated with modulation of HIF‐1α/VEGF signaling. In vivo, early hypoperfusion activated QKI5 and angiogenic pathways, followed by a gradual decline during sustained hypoperfusion.

**Conclusion:**

Progressive attenuation of the HCG11/QKI5‐mediated angiogenic program may underlie endothelial adaptive failure in cerebral hypoperfusion. Targeting this RNA‐regulatory axis may represent a potential therapeutic strategy to preserve vascular compensation and mitigate disease progression in ICAS.

## Graphical abstract

Chronic cerebral hypoperfusion induces endothelial hypoxic stress. During the early adaptive phase, the HCG11/QKI5 axis acts upstream of HIF‐1α/VEGF signaling to promote compensatory angiogenesis. With prolonged hypoperfusion, this regulatory pathway declines, leading to impaired vascular adaptation.

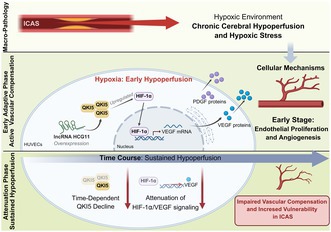

## 1 Introduction

Intracranial atherosclerotic stenosis (ICAS) is a leading cause of ischemic stroke and stroke recurrence worldwide [1]. Progressive arterial narrowing frequently results in chronic cerebral hypoperfusion [2], the severity of which depends on lesion characteristics and the capacity for vascular compensation [3]. Endothelial dysfunction is central to ICAS pathogenesis, and sustained hypoperfusion further imposes chronic hypoxic stress on cerebral microvessels, necessitating adaptive endothelial responses to maintain tissue perfusion [4, 5]. Concomitantly, hypoxia‐inducible factor‐1α (HIF‐1α) is a principal mediator of endothelial adaptation to hypoxia, coordinating transcriptional programs that promote angiogenesis and vascular remodeling [6]. Activation of downstream targets, including vascular endothelial growth factor (VEGF), contributes to compensatory neovascularization under reduced cerebral blood flow [7]. Nevertheless, the pathophysiology of ICAS remains complex, with additional regulatory mechanisms beyond the conventional HIF‐1α/VEGF pathway requiring further investigation.

Increasing evidence suggests that cerebrovascular diseases involve dynamic crosstalk among multiple cellular and systemic components rather than isolated vascular alterations. The neurovascular unit (NVU) integrates interactions between endothelial cells, neurons, glia, and perivascular elements to maintain cerebral homeostasis. In chronic cerebral hypoperfusion, these interactions extend beyond the brain through bidirectional communication with systemic metabolic, inflammatory, and vascular pathways [8, 9]. This multi‐level crosstalk evolves during disease progression, with early hypoperfusion triggering adaptive responses and prolonged stress leading to NVU disruption and impaired vascular compensation. Within this framework, circulating biomarkers provide a practical, minimally invasive approach to assessing hypoxia‐responsive and vascular signals. Rather than directly mirroring the spatial and cellular complexity of the cerebrovascular microenvironment, peripheral measurements represent integrated outputs of central and systemic responses shaped by NVU–systemic crosstalk. Recognizing this distinction is essential for interpreting circulating biomarker profiles in ICAS and linking them to underlying cerebrovascular processes.

Long non‐coding RNAs (lncRNAs) have emerged as important regulators of endothelial biology, influencing proliferation [10], migration [11], and angiogenic remodeling [12] under pathological conditions. Human leukocyte antigen complex group 11 (HCG11), a lncRNA located within the major histocompatibility complex region [13], exhibits context‐dependent roles across different diseases, behaving as a tumor suppressor in various cancers [14, 15, 16] and as an oncogene in others [17, 18, 19]. However, its role in cerebral endothelial adaptation to hypoperfusion remains poorly defined. HCG11 RNA‐binding protein Quaking (QKI) is an RNA‐binding protein of the STAR family that regulates RNA stability and post‐transcriptional gene expression [20]. Among its isoforms, QKI5 is preferentially expressed in endothelial cells and plays a critical role in vascular development and angiogenic regulation [21]. Notably, RNA‐binding proteins and lncRNAs are increasingly recognized as key mediators linking intracellular signaling with intercellular communication, providing a mechanistic bridge between hypoxia‐responsive pathways and NVU crosstalk.

In our previous studies, we demonstrated that the HCG11/QKI5 axis modulates endothelial function and angiogenic capacity in atherosclerotic plaques. HCG11 expression was reduced in endothelial cells derived from vulnerable plaques, while restoration of HCG11 promoted endothelial proliferation and angiogenesis through upregulation of QKI5, which in turn stabilizes HCG11, forming a reciprocal regulatory loop. These findings suggest that the HCG11/QKI5 axis may act as an upstream regulator of endothelial angiogenic responses [22].

Despite advances in medical therapy, ICAS remains associated with a high risk of recurrent ischemic stroke [23]. Enhancing early compensatory angiogenesis during chronic cerebral hypoperfusion therefore represents a critical therapeutic objective. However, whether the HCG11/QKI5 axis interacts with hypoxia‐inducible signaling pathways, including HIF‐1α/VEGF signaling, to regulate endothelial adaptation during cerebral hypoperfusion remains unclear, especially in the context of NVU–systemic crosstalk and its link to circulating biomarkers. In the present study, we investigated the role of the HCG11/QKI5 axis in endothelial hypoxic adaptation and its interaction with HIF‐1α/VEGF signaling using hypoxic endothelial cell models and a rat model of chronic cerebral hypoperfusion, while integrating clinical biomarker analysis. Elucidating these mechanisms may provide novel insights into the temporal regulation of angiogenic compensation and improve the interpretation of circulating biomarkers in ICAS‐associated chronic cerebral hypoperfusion.

## 2 Methods and Materials

### 2.1 Clinical Sample Collection

The samples were obtained from the database constructed in our previous study [24]. Seven patients each with symptomatic ICAS (sICAS) and asymptomatic ICAS (aICAS) were randomly selected depending on whether there had been an acute ischemic stroke or transient ischemic attack responsible for the stenotic‐side vessel. Additionally, seven control individuals without ICAS were matched at random based on the age range of the patients with ICAS in the database. Elbow venous blood samples from the participants were collected and tested within 15 min. Blood samples were stored frozen at −80°C. These samples were quantified by ELISA analysis. Table 1 lists the clinical characteristics of all participants.

**TABLE 1:** Demographic and clinical characteristics of patients with ICAS and control.

Characteristics	Groups			*p*
	sICAS^a^ (*n* = 7)	aICAS^b^ (*n* = 7)	Control (*n* = 7)	
Age (years)	53.14 ± 10.75	53.71 ± 10.29	52.29 ± 4.92	0.592
Male, *n* (%)	6 (85.71)	4 (57.14)	3 (42.86)	0.999
BMI^c^ (kg/m^2^)	26.08 ± 2.68	26.56 ± 3.53	26.97 ± 2.13	0.875
Hypertension, *n* (%)	3 (42.86)	4 (57.14)	2 (28.57)	> 0.999
Diabetes mellitus, no. (%)	2 (28.57)	0	1 (14.29)	> 0.999
Smoking, *n* (%)	2 (28.57)	2 (28.57)	1 (14.29)	> 0.999
Alcohol use, *n* (%)	3 (42.86)	3 (42.86)	2 (28.57)	> 0.999
Hyperlipidemia, *n* (%)	4 (57.14)	3 (42.86)	2 (28.57)	> 0.999
NIHSS score, median (IQR)	2 (0–7)	0 (0–1)	/	0.037
LDL‐C (mmol/L)	1.96 ± 0.75	2.59 ± 1.13	2.68 ± 0.82	0.258
HDL‐C (mmol/L)	0.92 ± 0.34	1.13 ± 0.20	1.25 ± 0.23	0.050
Triglycerides (mmol/L)	1.67 ± 0.51	1.42 ± 0.65	1.82 ± 0.67	0.429
Total Cholesterol (mmol/L)	3.62 ± 0.87	4.476 ± 1.86	4.43 ± 0.86	0.432

*Note:* Data were presented as means ± standard deviation.

^a^ Symptomatic intracranial atherosclerotic stenosis.

^b^ Asymptomatic intracranial atherosclerotic stenosis.

^c^ Body mass index.

Plasma HIF‐1α, VEGF, platelet‐derived growth factor‐AB (PDGF‐AB), platelet‐derived growth factor‐BB (PDGF‐BB), tumor necrosis factor α (TNFα), erythropoietin (EPO), Sirtuin 1 (SIRT1) were measured by commercial enzyme‐linked immunosorbent assay kits (RayBiotech, Atlanta, USA) following the manufacturer's specifications. Endothelin‐1 (ET‐1) and platelet endothelial cell adhesion molecule‐1 (CD31) were measured by commercial enzyme‐linked immunosorbent assay kits (Sabbiotech, MD, USA) following the manufacturer's specifications. All samples were measured in triplicate, and the mean was scored.

### 2.2 Cell Culture and Transfection

Human umbilical vein endothelial cells (HUVECs) were purchased from American Type Culture Collection (ATCC). HUVECs were seeded at 3 × 10^5^ cells/well in 6‐well plates until they reached a density of 70%–80% of confluence. In order to overexpress HCG11, the HCG11 genomic fragment was cloned by polymerase chain reaction (PCR) and then inserted into the pcDNA3.1 vector (HCG11‐oe). si‐QKI5 and the negative control vector were designed by Ribobio Biotechnology (Guangzhou, China). Those plasmids were separately transfected into cells utilizing Lipofectamine 3000 (Thermo Fisher Scientific, Waltham, MA, USA). After 48 h, cells were harvested for further analysis.

### 2.3 Cell Culture

**FIGURE 1:**
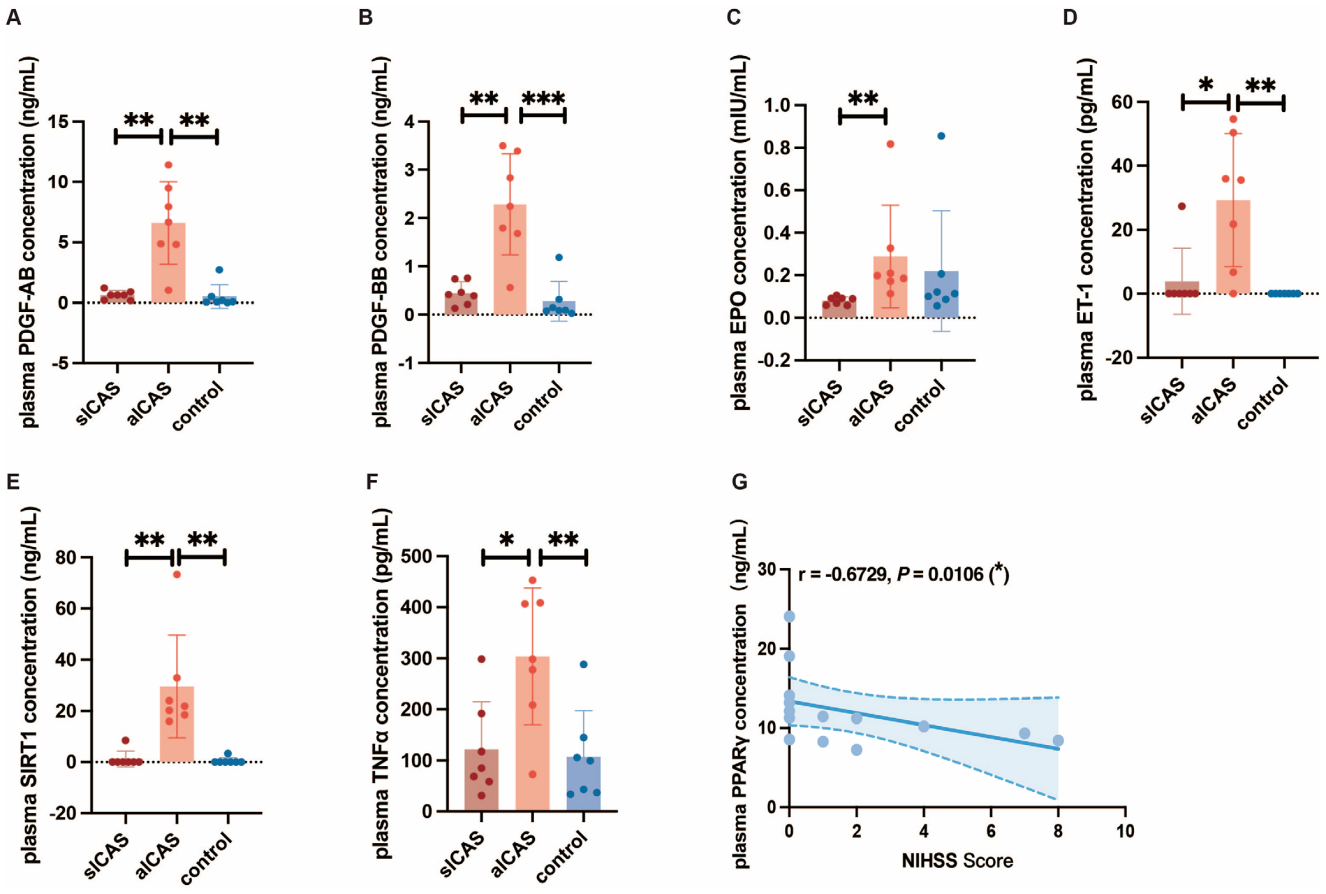
aICAS mediated inflammatory responses and oxidative stress, and induced neovascularization compensation. (A) plasma PDGF‐AB concentration. (B) plasma PDGF‐BB concentration. (C) plasma EPO concentration. (D) plasma ET‐1 concentration. (E) plasma SIRT1 concentration. (F) plasma TNFα concentration. (G) Spearman correlation analysis between NIHSS score of ICAS patients and plasma PPARγ. Data were presented as mean ± SD. *p < 0.05; **p < 0.01. sICAS, symptomatic intracranial atherosclerotic stenosis; aICAS, asymptomatic intracranial atherosclerotic stenosis.

HUVECs were divided into two groups: the oxygen–glucose deprivation (OGD) group and the normal oxygen and glucose (NOG) group. For the OGD group, the complete medium was replaced with a low‐glucose conditional medium (5.5 mM glucose) and incubated at 37°C with 1% O_2_ and 5% CO_2_ for 6 h. After 6 h of OGD, the medium was replaced with a normal medium and incubated at 37°C with 20% O_2_ and 5% CO_2_ for 18 h. NOG group was cultured in M199 medium containing 10% fetal bovine serum in a 20% O_2_, 5% CO_2_ humidified incubator at 37°C for 24 h (Figure 1A).

### 2.4 5‐Ethynyl‐2′‐Deoxyuridine (EdU) Assay

Cells with active DNA replication were labeled with the EdU Cell Proliferation Assay Kit (Ribobio, Guangzhou, China). According to the manufacturer's instructions as follows: cells were incubated with EdU for 2 h, following PBS washing, fixation, and permeation. Cells were stained with click chemistry operating solution for 30 min in the dark. Lastly, images were captured to calculate the proportion of EdU‐positive cells under a fluorescent stereomicroscope.

### 2.5 Matrigel‐Based Tube Formation Assay

Capillary‐like structure formation ability of HUVECs was assessed by Matrigel‐based tube formation assays. Aliquot 50 μL of growth factor‐reduced Matrigel into each well of the pre‐chilled 96‐well plate and polymerize at room temperature for 1 h on a level surface. 100 μL of the HUVECs suspension was dispensed and thoroughly mixed in the labeled wells of a 96‐well plate. The plates were incubated at 37°C, 5% CO_2_ for 4–6 h. Representative images of the capillary network were taken with a light microscope (magnification, ×400) and the tube lengths were measured using the Scion Image software.

### 2.6 Animals and Experimental Design

**FIGURE 2:**
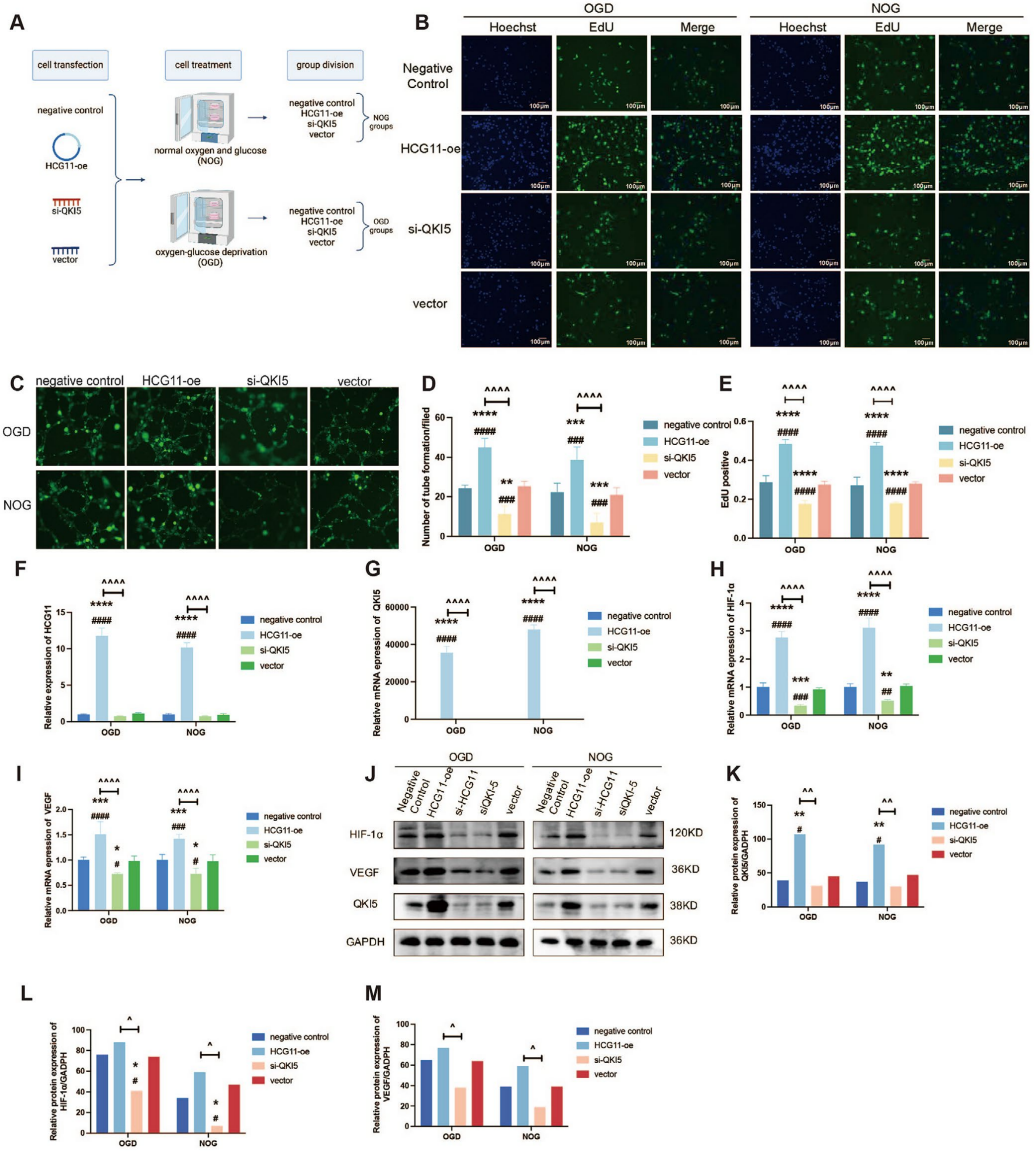
Upregulated HCG11/QKI5 activated HIF‐1α/VEGF pathway. (A) A schematic diagram of HUVECs experimental design. Firstly, HUVECs were transfected with different plasmids (HCG11‐oe, si‐QKI5 and vector). Then HUVECs were treated with OGD or NOG. Cells were divided into two groups of eight subgroups at last. OGD: Oxygen glucose deprivation. NOG: Normal oxygen glucose. HCG11‐oe: Overexpression. (B) The proliferation capacity of HUVECs was measured by the EdU assay, scale bar = 100 μm. (C) The angiogenesis ability of HUVECs was measured by the matrigel‐based tube formation assay. (D, E) The statistical data of tube formation assay and EdU assay respectively were presented as mean ± SD. (F–I) The expression of HCG11, QKI5 mRNA, HIF‐1α mRNA, VEGF mRNA were assessed by qPCR. Data were presented as mean ± SD. **p < 0.01 vs. negative control group; (J‐M) The protein expression of HCG11, QKI5, HIF‐1α, VEGF were assessed by Western Blot. Data were presented as mean. *p < 0.05 vs. negative control group; **p < 0.01 vs. negative control group; ****p < 0.0001 vs. negative control group. #*p* < 0.05 vs. vector group; ####*p* < 0.0001 vs. vector group. ^*p* < 0.05 vs. si‐QKI5 group; ^^*p* < 0.01 vs. si‐QKI5 group; ^^^^*p* < 0.0001 vs. si‐QKI5 group.

Adult male Sprague–Dawley rats weighing 230 ± 20 g were purchased from Charles River (Foshan, China). Prior to the experiments, rats were given a week of adaptive feeding. Rats were housed in a sound‐attenuated room under a 12‐h light/dark cycle at 22°C–24°C with unlimited access to food and water. A concerted effort was made to minimize both the number of animals tested and their agony. After surgical intervention, rats were divided into four groups of 18 rats per group, and from each group, six rats were sacrificed for blood and brain sample collection every 2 weeks (Figure 2A).

### 2.7 Surgical Intervention

#### 2.7.1 Unilateral Cerebroventricular Injection of Viral Vector

The rats were anesthetized with 2% sodium pentobarbital (3 mL/kg, i.p.), put in the stereotaxic apparatus over the heating pad and the rectal temperature was maintained at 37°C. After the skull was exposed, a small hole was drilled to reach the lateral cerebroventricle according to stereotaxic coordinates: −1.1 mm in the anteroposterior axis, ±1.5 mm in the mediolateral axis and −4.5 mm in the dorsoventral axis. According to the pre‐experiment results, the mortality rate of rats increased with the increase of adeno‐associated virus serotype 9 (AAV9) vector injection dose, therefore the final injection dose was set as 2 μL. 2 μL AAV9 solution expressing QKI5‐GFP or vector‐GFP (with a titre of 1 × 10^13^ vg/mL) was randomly injected into rats (*n* = 36).

#### 2.7.2 Bilateral Common Carotid Artery Occlusion (BCCAO)

Two weeks after the unilateral cerebroventricular injection, the rats were anesthetized again for BCCAO. During surgery, rats were placed on the heating pad to maintain the rectal temperature around 37°C until recovery from anesthesia. The bilateral common carotid arteries of rats were exposed through a median neck incision, the surrounding tissues were separated, and then ligated with surgical silk. The common carotid artery was initially ligated on one side, and then it was sutured layer by layer on the other side 20 to 30 min later. Only a midline cervical incision and suture were performed on the rats of the Sham group without BCCAO.

### 2.8 Immunohistochemistry Staining

Brain slices of rats were placed in citrate buffer solution at 95°C–99°C for 15 min. After washing and blocking, the slides were incubated with rat anti‐VEGF, anti‐HIF‐1α, or anti‐CD31 monoclonal antibodies overnight at 4°C. After PBS washing, slices were incubated with the secondary antibody for 1 h at 37°C, then stained with DAB chromogen substrate for 5 min in the dark. Finally, hematoxylin staining was performed for 4 min, and the sections were scanned using a Pannoramic MIDI automatic digital slide scanner (3D HISTECH, Budapest, Hungary).

### 2.9 Immunofluorescence

At various intervals following BCCAO induction, deeply anesthetized rats underwent perfusion with saline and 4% paraformaldehyde. Tissue samples were subsequently dehydrated using a sucrose gradient (20% and 30%) prepared in 4% PFA. Sagittal slices with a thickness of 20 μm were cut by a freezing microtome (Leica, Germany). For immunohistochemical staining, the sections were incubated overnight at 4°C with the following primary antibodies diluted in blocking solution: QKI5 (Rabbit, Proteintech, 13169‐1‐AP, 1:1000), HIF‐1α (Rabbit, Proteintech, 20960‐1‐AP, 1:500), and CD31 (Rabbit, Abcam, ab28364, 1:500). After removing the primary antibody and washing, a secondary antibody (Goat anti‐rabbit, Beyotime, A0208, 1:100) was applied and incubated for 1 h at room temperature. Nuclei were counterstained with DAPI for 15 min. Finally, the sections were mounted and scanned using a digital slide scanner (Pannoramic MIDI, 3D Histech, Hungary), and images were analyzed with the corresponding slide viewer software (Case Viewer 2.3, 3D Histech, Hungary).

To evaluate the distribution of AAV9‐mediated QKI5 overexpression, triple‐stained sections (QKI5/CD31/DAPI) were analyzed using QuPath 0.7.0 (QuPath software, Belfast, UK) [25]. QKI5‐positive (QKI5^+^) cells were quantified separately in cerebrovascular endothelial (CD31^+^) and non‐endothelial (CD31^−^) populations. Automated cell detection was applied to calculate the proportion of QKI5^+^ cells normalized to total DAPI^+^ nuclei per cell type.

### 2.10 Western Blot

HUVECs and rat brain tissue samples were collected, added RIPA lysate, and centrifuged. Supernatant proteins were extracted, and the concentration was determined by the BCA method. After SDS‐PAGE gel electrophoresis (80 V, 120 min), the total proteins were transferred to PVDF membranes, which were blocked with 5% skimmed milk at room temperature for 1 h, and then incubated with diluted primary antibody (HUVECs: HIF‐1α, 20960‐1‐AP, Proteintech, Wuhan, China; VEGF, 19003‐1‐AP, Proteintech, Wuhan, China; QKI5, 13169‐1‐AP, Proteintech, Wuhan, China; GAPDH, AF5003, Goodhere, Hangzhou, China. Rats: VEGF, 19003‐1‐AP, Proteintech, Wuhan, China; HIF‐1α, bs‐20399R, Bioss Inc., Beijing, China; CD31, sc‐3767, Santa Cruz Biotechnology, Shanghai, China; STAT‐3, 60199‐1‐IG, Proteintech, Wuhan, China; PDGF‐AB, ab9701, Abcam, Shanghai, China; PDGF‐BB, ab178409, Abcam, Shanghai, China; GAPDH, AF5003, Goodhere, Hangzhou, China) overnight at 4°C. The membranes were washed three times with TBST, incubated with secondary antibodies (horseradish peroxidase, A0208, A0216, Beyotime Biotechnology, Shanghai, China) for 1 h, and washed with TBST again. The membranes were incubated with the ECL reaction solution for 2 min before being imaged to expose the protein bands. Finally, ImageJ software was used to calculate the protein's gray value.

### 2.11 Quantitative Real‐Time PCR (qPCR)

Total RNA was extracted from HUVECs or rat brain tissues with Trizol solution, and then the RNA was reverse transcribed to complementary DNA (cDNA) using a GoScript kit (Promega, Beijing, China). cDNA, primers and AceQ qPCR SYBR Green Master Mix (Vazyme, Nanjing, China) were mixed and subjected to PCR amplification cycles on a Real‐Time PCR instrument (ABI, Alexandria, USA). qPCR program was set to 95°C for 5 min, followed by 40 cycles of 95°C for 10 s and 60°C for 20 s. Primer sequences were as follows:

HUVECs: HCG11, Forward, 5′‐GGAAAGAGGTTGCCGACGTA‐3′, and Reverse, 5′‐AGTGAGGAAGCGAATGCACA‐3′. QKI5, Forward, 5′‐CCTGATGCAGCTGATGAACG‐3′, and Reverse, 5′‐GTCCCACAGCATCAGGCAAT‐3′. HIF‐1α, Forward, 5′‐CAGAATGGAATGGAGCAAAAG‐3′, and Reverse, 5′‐TGGTCAGCTGTGGTAATCCA‐3′. VEGF, Forward, 5′‐AGGGCAGAATCATCACGAAGT‐3′, and Reverse, 5′‐AGGGTCTCGATTGGATGGCA‐3′. GAPDH, Forward, 5′‐TCATTGACCTCAACTACATGG‐3′, and Reverse, 5′‐TCGCTCCTGGAAGATGGTG‐3′.

Rats: QKI5, Forward, 5′‐TGGAAACGAAGGAGAAGCCG‐3′ and Reverse, 5′‐GTGGTTGAAGATCCCGCAGA‐3′. HIF‐1α, Forward, 5′‐TGGAAGCACTAGACAAAGCTCA‐3′, and Reverse, 5′‐TTGACCATATCGCTGTCCAC‐3′. VEGF, Forward, 5′‐GCAGCGACAAGGCAGACTAT‐3′, and Reverse, 5′‐GAGGGAGTGAAGGAGCAACC‐3′. STAT‐3, Forward, 5′‐ACCAACGACCTGCAGCAATA‐3′, and Reverse, 5′‐ACACTCCGAGGTCAGATCCA‐3′. PDGF‐AA, Forward, 5′‐AGGACGCGTAGAACAATCGG‐3′, and Reverse, 5′‐GGAGATTCACCGGATGGCTT‐3′. PDGF‐BB, Forward, 5′‐TGAAGACGAACCATCGGCTG‐3′, and Reverse, 5′‐GGGGACTCCAACCTCAGAGA‐3′. GAPDH, Forward, 5′‐TCATTGACCTCAACTACATGG‐3′, and Reverse, 5′‐TCGCTCCTGGAAGATGGTG‐3′.

GAPDH was used as a housekeeping gene. Relative levels of gene expression were calculated by the Delta Delta Ct method.

### 2.12 Statistical Analysis

All statistical analyzes were performed using GraphPad Prism 10.0 (GraphPad Software, CA, USA), with a significance level set at *p* < 0.05 (two‐tailed). Continuous variables were initially evaluated for normality and variance homogeneity. If the assumptions were met, one‐way analysis of variance (ANOVA) was used for overall comparisons, followed by Tukey's post hoc test for pairwise comparisons. If the assumptions were not met, Kruskal‐Wallis *H* test was applied, followed by Dunn's post hoc test for pairwise comparisons. Spearman correlation analysis was used for correlation analysis. Categorical variables were analyzed using Fisher's exact test, with Bonferroni correction for multiple comparisons. For changes within the same group at different time points, two‐way repeated measures ANOVA was used. Data are presented as mean ± standard deviation or median (interquartile range), as appropriate.

## 3 Results

### 3.1 Early Asymptomatic ICAS Is Associated With Activation of Hypoxia‐Related Angiogenic Responses

There were no statistically significant differences in the clinical and demographic features among sICAS patients, aICAS patients, and controls (Table 1, *p* > 0.05).

To determine whether chronic cerebral hypoperfusion is associated with systemic hypoxia‐responsive activation, circulating biomarkers in participants were analyzed. The aICAS group showed significantly higher levels of PDGF‐AB, PDGF‐BB, EPO, ET‐1, SIRT1, and TNF‐α compared to the sICAS group, and except for EPO, these indicators were also significantly higher than the control group (Figure 1A–F). In contrast, the expression levels of pro‐angiogenic or inflammatory factors in sICAS patients were similar to those in the control group, with no statistically significant changes. These findings indicate that early‐stage ICAS (asymptomatic ICAS) is characterized by an active hypoxia‐responsive and pro‐angiogenic state. Given that NIHSS scores differ inherently between sICAS and aICAS patients, correlation analyzes were performed to evaluate the potential confounding effects of clinical severity. Among the biomarkers examined, PPARγ levels showed a significant negative correlation with NIHSS scores (Figure 1G), indicating that lower PPARγ levels were associated with greater neurological impairment.

### 3.2 The HCG11/QKI5 Axis Regulates Endothelial Proliferation and Angiogenesis In Vitro

To investigate the contribution of the HCG11/QKI5 axis to endothelial angiogenic activity, HUVECs were treated with HCG11 overexpression or QKI5 knockdown under normoxic and OGD conditions. HCG11 overexpression significantly enhanced endothelial proliferation and tube formation, whereas QKI5 knockdown significantly suppressed these responses (Figure 2B–E). OGD alone did not induce significant angiogenic activation. These results demonstrated that endothelial angiogenic responses under hypoxic stress required regulatory modulation via the HCG11/QKI5 axis.

### 3.3 The HCG11/QKI5 Axis Modulates HIF‐1α/VEGF Signaling in Endothelial Cells

To determine whether the HCG11/QKI5 axis influences canonical hypoxia‐inducible signaling, expression levels of HIF‐1α and VEGF were assessed following HCG11 overexpression or QKI5 knockdown. HCG11 overexpression increased, whereas QKI5 knockdown decreased HIF‐1α and VEGF expression at both the mRNA and protein levels (Figure 2F–M). These coordinated changes demonstrate that the HCG11/QKI5 axis modulates endothelial hypoxia‐responsive angiogenic signaling.

### 3.4 Chronic Cerebral Hypoperfusion Induces Transient Endothelial Activation of QKI5‐Driven Angiogenic Signaling In Vivo

**FIGURE 3:**
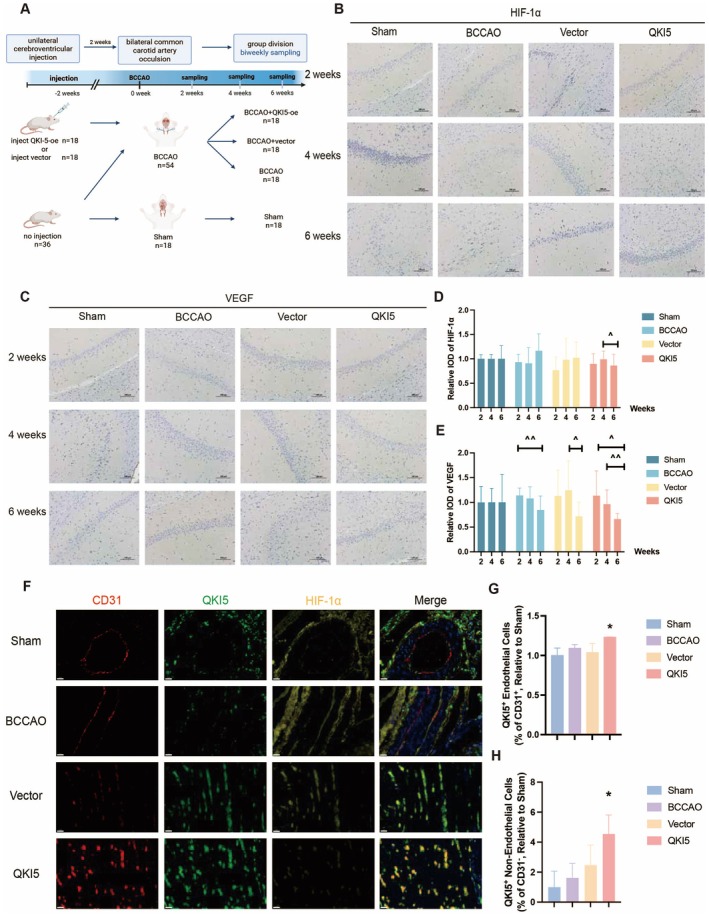
BCCAO induced cerebral hypoperfusion activating hypoxia‐related pathways of endothelial cells in the rat brain. (A) A schematic diagram of BCCAO rat experimental design. Before the operation, 36 rats were respectively injected with either QKI5‐oe or vector into the unilateral ventricle. Two weeks later, those rats received injection and 18 rats without injection were subjected to BCCAO surgery. While rats of Sham group (*n* = 18) received only midline cervical incision and suture. Therefore rats were divided into four groups: Sham, BCCAO, BCCAO+QKI5‐oe and BCCAO + Vector. After BCCAO, blood and brain samples were collected from six rats per group biweekly (Created in https://BioRender.com). BCCAO: Bilateral common carotid artery occlusion. (B, C) Immunohistochemistry was performed to track HIF‐1α/VEGF protein levels in the rat brain from 2 to 6 weeks post‐BCCAO. Scale bar = 50 μm. (D, E) The statistical data of immunohistochemistry staining were presented as mean ± SD. ^*p* < 0.05; ^^*p* < 0.01; (F) Multiplex immunofluorescence of CD31‐labeled endothelial cells (yellow), QKI5 (green) and HIF‐1α (red) within the cortex in the Sham, BCCAO, Vector and QKI5 groups at 2 weeks after BCCAO. Scale bar = 20 μm. (G, H) Cell‐type distribution for AAV9‐mediated QKI5 overexpression was assessed at 2 weeks after BCCAO. (G) Quantification of QKI5‐positive endothelial cells (CD31^+^), expressed as a percentage of total endothelial cells and normalized to the sham group. (H) Quantification of QKI5‐positive non‐endothelial cells (CD31^−^), expressed as a percentage of total non‐endothelial cells and normalized to the sham group. Data were presented as mean ± SD. *p < 0.05 vs. Sham group.

To investigate endothelial hypoxic adaptation in vivo, a BCCAO rat model was established. Immunohistochemical analysis demonstrated that the expression of HIF‐1α and VEGF in cerebral microvessels gradually declined with prolonged hypoperfusion (Figure 3B–E). Immunofluorescence staining revealed prominent co‐localization of QKI5 and HIF‐1α with the endothelial marker CD31 in cortical microvessels at 2 weeks after BCCAO (Figure 3F). These results indicate that chronic hypoperfusion activates endothelial hypoxia‐responsive signaling pathways in vivo, with QKI5 induction occurring early during vascular adaptation. To evaluate the in vivo distribution of AAV9‐mediated QKI5 overexpression, we quantified QKI5‐positive (QKI5^+^) cells in cerebrovascular endothelial (CD31^+^) and non‐endothelial (CD31^−^) populations using triple immunofluorescence staining (CD31/QKI5/DAPI) at 2 weeks after BCCAO (Figure 3G,H). In endothelial cells, the proportion of QKI5^+^ cells was significantly increased in the QKI5 group compared with the sham group (Figure 3G), indicating that AAV9 delivery enhances QKI5 expression in cerebrovascular endothelium. A similar but more pronounced increase was observed in the non‐endothelial cell population (Figure 3H). Overall, intracerebroventricular AAV9 delivery increased QKI5 expression across multiple brain cell types, including endothelial cells, with a stronger effect in non‐endothelial populations. This distribution pattern is consistent with the known broad cellular tropism of AAV9 and suggests that the in vivo overexpression model reflects system‐level modulation rather than endothelial‐specific targeting.

### 3.5 Sustained Hypoperfusion Leads to Time‐Dependent Attenuation of QKI5‐Driven Pro‐Angiogenic Signaling

**FIGURE 4:**
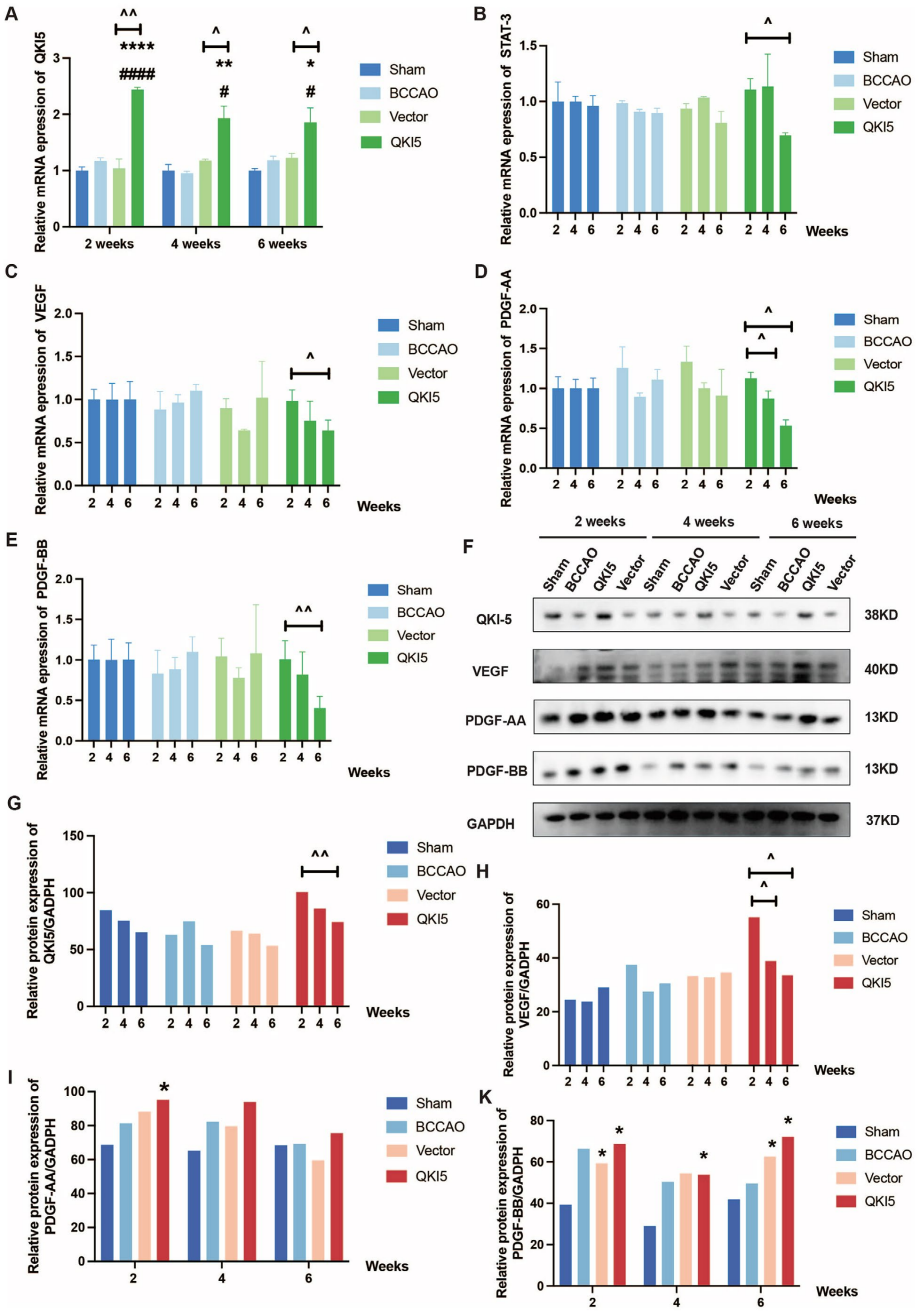
Hypoxia‐induced proangiogenic factors were significantly reduced with long‐term effect of cerebral hypoperfusion. (A–E) The mRNA expression of QKI5, STAT3, VEGF, PDGF‐AA, PDGF‐BB were assessed by qPCR. Data were presented as mean ± SD. *p < 0.05 vs. Sham group; **p < 0.01 vs. Sham group; ****p < 0.0001 vs. Sham group. #*p* < 0.05 vs. BCCAO group; ####*p* < 0.0001 vs. BCCAO group. ^*p* < 0.05 vs. Vector group; ^^*p* < 0.01 vs. Vector group. (F–K) The protein expression of QKI5, PDGF‐AA, VEGF, PDGF‐BB were assessed by Western Blot. Data were presented as mean. *p < 0.05 vs. Sham group. ^*p* < 0.05 vs. Vector group; ^^*p* < 0.01 vs. Vector group.

Lateral ventricular injection significantly increased cerebral QKI5 mRNA expression (Figure 4A). Notably, in the QKI5‐overexpression group, mRNA levels of QKI5 and downstream pro‐angiogenic factors, including STAT3, VEGF, PDGF‐AA, and PDGF‐BB, declined progressively with prolonged hypoperfusion (Figure 4B–E). This temporal reduction was not observed in sham, BCCAO‐only, or vector‐treated groups. Consistent with transcriptional alterations, protein levels of QKI5 and VEGF significantly decreased over time in the QKI5‐overexpression group (Figure 4G,H), whereas, compared with the sham group, PDGF‐AA of the QKI5‐overexpression group was significantly elevated at 2 weeks after BCCAO (Figure 4I). Moreover, as a chronic hypoxia‐responsive factor, PDGF‐BB in the QKI5‐overexpression group was significantly higher than that in the sham group from 2 to 6 weeks, indicating the QKI5 axis may accelerate the hypoxia adaptation process in rats with chronic cerebral hypoperfusion (Figure 4K).

These findings indicate that although QKI5 activation enhances early hypoxia‐induced angiogenic signaling, sustained cerebral hypoperfusion progressively attenuates this compensatory response.

## 4 Discussion

In the present study, we investigated endothelial adaptive responses to chronic cerebral hypoperfusion secondary to ICAS, with particular attention to the regulatory role of the HCG11/QKI5 axis and its interaction with hypoxia‐inducible signaling. By integrating clinical observations with in vitro hypoxic endothelial models and an in vivo BCCAO rat model, it has been demonstrated that the HCG11/QKI5 axis was engaged in early hypoxia‐responsive angiogenic programs and appears to modulate HIF‐1α/VEGF signaling during cerebral hypoperfusion. HCG11 overexpression enhanced endothelial proliferation and angiogenesis in parallel with increased HIF‐1α and VEGF expression, whereas suppression of HCG11 or QKI5 attenuated hypoxia‐responsive angiogenic activity. In vivo, early QKI5 upregulation was associated with increased pro‐angiogenic factor expression, followed by a time‐dependent decline with prolonged hypoperfusion. Together, these findings identify the HCG11/QKI5 axis as a key modulator of early endothelial compensatory neovascularization in response to chronic cerebral hypoperfusion.

Circulating biomarker analysis showed that patients with asymptomatic ICAS exhibited significantly higher levels of pro‐angiogenic, inflammatory, and oxidative stress–related markers compared with symptomatic patients and controls. Asymptomatic ICAS is associated with reduced cerebral perfusion and oxygen utilization [26], which likely triggers adaptive vascular responses to preserve blood flow [4, 27]. In contrast, the relative absence of such responses in symptomatic ICAS suggests exhaustion of endogenous compensatory mechanisms, aligning with prior findings of temporally limited angiogenic and collateral circulation responses during prolonged cerebral ischemia [28, 29]. These findings support a stage‐dependent model in which asymptomatic ICAS represents an early compensatory phase characterized by active vascular adaptation, whereas symptomatic ICAS reflects a later stage marked by failure or exhaustion of these mechanisms. Notably, PDGF‐AB and PDGF‐BB were preferentially elevated in asymptomatic patients, whereas HIF‐1α and VEGF showed no significant differences. Since PDGF signaling promotes perivascular stabilization, vascular smooth muscle cell recruitment, and maturation of nascent vessels [30, 31, 32], the observed increase in PDGF‐AB and PDGF‐BB suggests that early compensatory adaptation may rely more on PDGF‐mediated vascular stabilization and remodeling, which can precede or complement HIF‐1α/VEGF‐driven angiogenesis during hypoperfusion. Although the possibility that ex vivo platelet hyperreactivity increased PDGF expression cannot yet be ruled out, several observations support its association with compensatory adaptation. In particular, elevated PDGF has been observed in asymptomatic ICAS, a phase characterized by chronic but compensatory hypoperfusion rather than acute thrombotic activity, and elevated PDGF occurred concomitantly with coordinated changes in other hypoxia‐responsive and vascular‐related factors. Meanwhile, Arun Subramanian et al. pointed out that excessive platelet activation was present in patients with recently symptomatic carotid stenosis, which was significantly higher than in healthy controls and patients with asymptomatic carotid stenosis [33]. These findings suggest that PDGF may serve as a marker of early vascular remodeling in hypoperfusion. In parallel, the inverse correlation between NIHSS scores and PPARγ levels indicates that reduced PPARγ activity is associated with more severe neurological impairment. Given its established role in maintaining vascular homeostasis, protecting endothelium and suppressing inflammation [34, 35, 36], circulating PPARγ may reflect disease severity and endothelial vulnerability rather than compensatory adaptation [37, 38]. Together, these findings support the notion that distinct circulating biomarkers capture different aspects of ICAS progression, with PDGF reflecting stage‐dependent vascular compensation and PPARγ indicating disease severity. Such distinctions may help explain the heterogeneity of biomarker profiles in ICAS and highlight the importance of integrating multiple biological axes when assessing disease progression.

At the cellular level, our in vitro experiments indicate that the HCG11/QKI5 axis contributes to endothelial proliferation and angiogenic activity under hypoxic circumstances. Overexpression of HCG11 promoted endothelial proliferation and tube formation, whereas QKI5 knockdown exerted inhibitory effects [22]. These functional changes were accompanied by parallel alterations in HIF‐1α and VEGF expression, supporting a modulatory role of the HCG11/QKI5 axis upstream of conventional hypoxia‐responsive angiogenic signaling. Notably, hypoxia‐dependent regulation of QKI appears to be context‐specific. In head and neck squamous cell carcinoma, HIF‐1α‐induced microRNA‐5100 suppresses QKI expression and promotes tumor progression [39], whereas in cerebral endothelial cells exposed to chronic hypoperfusion, our data indicate that QKI5 is transiently upregulated and associated with early angiogenic adaptation. Therefore, these findings suggest that QKI functions as a hypoxia‐responsive regulatory node whose effects are determined by cellular environment. Besides, only OGD treatment was insufficient to induce robust angiogenic responses, underscoring the importance of regulatory pathways in shaping endothelial adaptation to hypoxic stress.

The enhancement of HIF‐1α expression by HCG11 under hypoxic conditions supports a role for long non‐coding RNA–mediated regulation in fine‐tuning endothelial hypoxia responses [40]. Given the established function of QKI5 as an RNA‐binding protein involved in post‐transcriptional regulation, it is plausible that QKI5 influences hypoxia‐inducible signaling through effects on RNA stability or translational control [41, 42, 43]. Although these molecular interactions were not directly examined, coordinated regulation at both mRNA and protein levels suggests functional crosstalk between the HCG11/QKI5 axis and HIF‐1α–dependent pathways.

Findings from the BCCAO model further emphasize the temporal dynamics of endothelial adaptation in response to chronic hypoperfusion. In the early phase, QKI5 overexpression was associated with HIF‐1α co‐localization and increased expression of downstream pro‐angiogenic factors, including VEGF and PDGFs, indicating activation of a compensatory angiogenic program. However, with prolonged hypoperfusion, this response progressively declined, as reflected by reduced QKI5‐associated signaling and attenuation of VEGF and PDGF expression, suggesting a time‐dependent loss of angiogenic responsiveness. This attenuation likely reflects a transition from an initially adaptive to a maladaptive endothelial state. Sustained hypoxic stress may first impose cumulative metabolic and oxidative burden, which in turn disrupts hypoxia‐responsive signaling cascades. This could lead to reduced stability or activity of HIF‐1α as well as impaired transcriptional output of downstream angiogenic genes [44, 45]. In parallel, the diminished responsiveness despite continued QKI5 overexpression suggests that the post‐transcriptional regulatory capacity of QKI5 may become functionally constrained under chronic hypoxic conditions, potentially due to alterations in RNA stability, translational efficiency, or RNA–protein interaction dynamics [20, 43, 46, 47]. Moreover, prolonged hypoperfusion is known to induce endothelial dysfunction and microvascular rarefaction [48, 49, 50], which may further limit the cellular capacity to respond to pro‐angiogenic cues. Together, these processes form a feed‐forward loop in which impaired signaling and structural vascular changes reinforce each other, ultimately leading to attenuation of angiogenic adaptation. This pattern is consistent with the clinically observed reduction of compensatory capacity in symptomatic ICAS. In general, our findings support a model in which QKI5‐driven angiogenic responses are temporally restricted, with early activation followed by progressive attenuation under sustained hypoperfusion, reflecting a transition from adaptive vascular remodeling to functional exhaustion.

The stage‐dependent regulation of PDGF signals observed in vivo further demonstrates its involvement in early vascular remodeling rather than a passive or non‐specific increase. Given its well‐established role in perivascular stabilization and maturation of nascent vessels [32, 51], PDGF may contribute to early compensatory vascular adaptation during hypoperfusion, potentially preceding or complementing canonical HIF‐1α/VEGF‐driven angiogenesis. To further interpret these in vivo findings, we evaluated the cellular distribution of AAV9‐mediated QKI5 overexpression and found that QKI5 expression was significantly increased in both cerebrovascular endothelial and non‐endothelial cell populations, with a more pronounced elevation in the latter. This pattern is consistent with the known broad cellular tropism of AAV9 [52], indicating that intracerebroventricular delivery results in systemic rather than endothelial‐specific modulation. Importantly, despite this lack of strict specificity, QKI5 expression was significantly elevated in endothelial cells, supporting the relevance of this model for investigating endothelial responses under hypoperfusion. However, concurrent upregulation in non‐endothelial cells suggests that the observed in vivo effects may reflect integrated responses within the neurovascular unit, as neurons and glial cells can modulate vascular function through paracrine and metabolic interactions.

Collectively, these findings point to a scenario in which the HCG11/QKI5 axis participates in early‐stage endothelial hypoxic adaptation by promoting angiogenic remodeling and coordinating multicellular responses within the neurovascular unit. As hypoperfusion persists, this compensatory signaling diminishes, potentially contributing to insufficient collateral development and increased vulnerability to ischemic injury. Several limitations should be noted. The clinical sample size was relatively limited. Although our data are consistent with a potential modulatory role of the HCG11/QKI5 axis in HIF‐1α/VEGF signaling, the precise molecular mechanisms such as effects on RNA stability, translational regulation, or direct RNA–protein interactions were not directly investigated and need further study. In addition, intracerebroventricular AAV9 delivery resulted in QKI5 overexpression across multiple cell types, and circulating PDGF levels may be influenced, in part, by ex vivo platelet hyper‐reactivity; therefore, they may not exclusively reflect vascular‐derived signals. Future studies employing endothelial‐specific genetic strategies and platelet‐controlled analyzes will be required to further dissect cell‐type–specific mechanisms and to determine the long‐term functional consequences of modulating this pathway on cerebral perfusion and neurological outcomes.

Taken together, our findings indicate that activation of the HCG11/QKI5 axis constitutes an early adaptive endothelial response to chronic cerebral hypoperfusion rather than a sustained pro‐angiogenic program. The time‐dependent attenuation of QKI5 signaling highlights the limited durability of endogenous neovascularization compensation. Notably, concordant alterations in vascular tissue and circulating profiles suggest that HCG11/QKI5 may serve as a systemic indicator of cerebrovascular hypoxic status, with potential utility for disease staging and monitoring.

## 5 Conclusion

This study demonstrates that the HCG11/QKI5 axis is activated during chronic cerebral hypoperfusion and contributes to early endothelial angiogenic adaptation. The time‐dependent attenuation of this response suggests that endogenous neovascularization is insufficient to sustain long‐term perfusion compensation. Circulating HCG11/QKI5 may serve as candidate biomarkers reflecting cerebrovascular hypoxic adaptation and disease stage, with potential utility for risk stratification and therapeutic monitoring in chronic hypoperfusion‐related disorders.

## Author Contributions

X.J. contributed to writing – original draft preparation. R.L. and F.Z. were involved in methodology. L.L. and L.W. were responsible for data curation. X.W. and F.Y. were involved in validation. J.L. and X.G. were responsible for writing – review and editing. X.J. contributed to project administration. All the authors have read and agreed to the published version of the manuscript.

## Funding

This work was supported by National Natural Science Foundation of China (Grant 82171302), National Science and Technology Major Project (Grant 2023ZD0503806) and Capital's Funds for Health Improvement and Research (Grant 2024‐1‐2011).

## Ethics Statement

The study was in accordance with the Declaration of Helsinki and approved by the Ethics Committee of Xuanwu Hospital, Capital Medical University (protocol code: 2018071, KS2022157‐1 and date of approval: September 13th, 2022). And written informed consent has been obtained from the participants to participate in the study and to publish this paper.

## Conflicts of Interest

The authors declare no conflicts of interest.

## Data Availability

The data that support the findings of this study are available from the corresponding author upon reasonable request.
